# Infant Safe Sleep Practices as Portrayed on Instagram: Observational Study

**DOI:** 10.2196/27297

**Published:** 2021-11-15

**Authors:** Samuel Chin, Rebecca Carlin, Anita Mathews, Rachel Moon

**Affiliations:** 1 Department of Pediatrics University of Virginia School of Medicine Charlottesville, VA United States; 2 Department of Pediatrics Columbia University School of Medicine New York, NY United States; 3 Children's National Medical Center Washington, DC United States

**Keywords:** sleep position, bed-sharing, social norms, social media, safe sleep, bedding

## Abstract

**Background:**

Parenting practices are highly influenced by perceived social norms. Social norms and American Academy of Pediatrics (AAP) guidelines for infant safe sleep practices are often inconsistent. Instagram has become one of the most popular social media websites among young adults (including many expectant and new parents). We hypothesized that the majority of Instagram images of infant sleep and sleep environments are inconsistent with AAP guidelines, and that the number of “likes” for each image would not correlate with adherence of the image to these guidelines.

**Objective:**

The objective of this study was to determine the extent of adherence of Instagram images of infant sleep and sleep environments to safe infant sleep guidelines.

**Methods:**

We searched Instagram using hashtags that were relevant to infant sleeping practices and environments. We then used an open-source web scraper to collect images and the number of “likes” for each image from 27 hashtags. Images were analyzed for adherence to AAP safe sleep guidelines.

**Results:**

A total of 1563 images (1134 of sleeping infant; 429 of infant sleep environment without sleeping infant) met inclusion criteria and were analyzed. Only 117 (7.49%) of the 1563 images were consistent with AAP guidelines. The most common reasons for inconsistency with AAP guidelines were presence of bedding (1173/1563, 75.05%) and nonrecommended sleep position (479/1134, 42.24%). The number of “likes” was not correlated with adherence of the image to AAP guidelines.

**Conclusions:**

Although individuals who use Instagram and post pictures of sleeping infants or infant sleep environments may not actually use these practices regularly, the consistent portrayal of images inconsistent with AAP guidelines reinforces that these practices are normative and may influence the practice of young parents.

## Introduction

Studies have investigated the effect that personal social networks (ie, individuals with whom one has personal relationships, social interactions, or both) can have on certain adult health behaviors, such as diet, nutrition, smoking, and obesity [1-3]. These social networks, which traditionally have been largely face-to-face, can also influence parenting practices, such as breastfeeding initiation and continuation [4-8] and vaccination [9]. Data suggest that online social networks, such as Facebook, Instagram, and others, are increasingly important influences on parental practice, including parental smoking cessation [10] and child nutrition [11,12].

It is likely that much of the influence from social networks (face-to-face or online) is derived from the network members providing their opinions about what behaviors and practices are expected and acceptable [13,14]. One then perceives these behaviors and practices to be normative behavior (everyone does this) and strives to adhere to these social norms to avoid judgment and reproach [2,3,15-22]. These social norms can be very powerful. Studies have found social norms to be a key variable mediating the association between maternal education and certain infant care practices [23] and the association between maternal country of birth and breastfeeding [24]. When social norms are contrary to evidence-based guidelines, they can negatively impact health.

One area in which there is often much discrepancy between social norms and evidence-based guidelines is the area of infant safe sleep practices [25]. Certain sleep practices, including nonsupine sleep position, use of soft bedding, soft sleep surfaces, and bed-sharing, are associated with increased risk for sudden unexpected infant death (SUID) [26] and the American Academy of Pediatrics (AAP) has published evidence-based guidelines for infant safe sleep [27] to reduce the risk of SUID. However, although there may be ample public health guidelines and education in the health care professional’s office before and after birth, other influences outside the health care setting may have an even stronger impact on sleep practices [28-31], and inconsistent messages about where and how the infant should sleep are associated with nonadherence to safe sleep guidelines [32].

Given that SUID rates in the United States have not declined since 2000 [33], and rates of nonsupine positioning [34], bed-sharing [35,36], and use of soft bedding [37] have not decreased, increased attention has been paid to the importance of changing social norms surrounding these practices. One randomized controlled trial of safe sleep video messages sent to new mothers by SMS text message or email resulted in improvements in safe sleep practices, and demonstrated that these improvements were mediated in part by changes in the mothers’ perceptions of the social norms surrounding the particular practices [38].

Media has traditionally been very one of the most influential factors in establishing societal and perceived social norms [39,40]. One qualitative study of new mothers found that images of sleeping infants and infant sleep environments, as found in photographs, books, television, and the internet, were one of the most consistent influences on their decisions about how infants slept at home [41]. However, these images are often inconsistent with safe infant sleep guidelines [42-44]. The power of these images may be increasing as marketing and social networking have come together synergistically to more effectively reach and influence target audiences. Today, nearly anyone can share personal experiences by writing reviews or commenting on and rating experiences. These interactions are highly influential in decisions regarding product purchases [45]. While the effect of these images on decisions regarding product purchases and parenting practices may differ, product purchases (eg, cribs, soft bedding) directly impact on infant sleep practices. Because many products marketed or used for infant sleep do not in fact meet federal safety standards [46] and are not safe for infant sleep, product selection and thus marketing are relevant to increasing safe sleep practices. Parents may be persuaded to purchase these products because they infer from social media that these products are not risky [47] and that use of these products is normative and acceptable infant care practice [48]. Additionally, the structure of social media allows one to selectively view specific advertisers or personalities by “following” them. Similar products or persons are then suggested based on algorithms utilizing one’s past online searches. While “following” specific advertisers or personalities creates some self-selection and selection bias regarding what is seen, an algorithm can be triggered by an online search that merely suggests that one is pregnant or has a new infant. This reinforcing nature of social media [49] can potentially make any exposure to certain practices or ideas even more powerful.

Instagram, which is mainly a photo-sharing application (app), has become one of the most popular social media apps/websites among young adults (including many expectant and new parents); among the >1 billion monthly active users [50], 56.3% of users are women, and those aged 25-34 years comprise the largest user group [51]. Further, Instagram is the most popular social media platform for teenagers, with 72% of them being active users [52]. This app, like many others, is designed so that users spend time on the app, and there has been growing concern about the phenomenon of Instagram “addiction.” One study found that 2 major needs may contribute to Instagram addiction: recognition (need for admiration from others through Instagram posts) and social (use of Instagram to share views and maintain contact with others) [53]. However, the vast majority of Instagram users do not have high levels of Instagram addiction [54].

Instagram users post photos or videos of content, often with a hashtag (a word or phrase preceded by the # symbol) frequently attached. Tagging with a hashtag allows others to easily find other messages or images that have a similar theme or content. Instagram users can also indicate that they “like” a photo by clicking on a heart icon. The number of “likes” for a photo implies the degree of social endorsement [55]. One small study found that adolescents who viewed photos were more likely to “like” photos with many “likes.” This study also found, using functional magnetic resonance imaging, that viewing photos with many “likes” stimulated neural regions associated with social cognition, reward learning, imitation, and attention [55]. Thus, “likes” can act as a form of peer influence and create the perception of normative behavior.

According to surveys conducted by Instagram’s parent company (Facebook), 78% and 74% of surveyed Instagram users, respectively, state that they perceive products or product brands viewed on Instagram to be popular and relevant, and 81% use Instagram to help them discover or research products or services [56]. Nearly half reported having made a purchase after seeing a product or service on Instagram. Largely because of Instagram’s popularity among potential consumers, nearly half of businesses are active on Instagram [56].

Although Instagram images provide only a snapshot of a single point in time, and although we acknowledge that these images may not accurately reflect how and where the infants portrayed in the images actually sleep, we aimed in this study to determine what proportion of images of sleeping infants and infant sleep environments were consistent with infant safe sleep guidelines. Because hashtags may be used to search for specific content, we wanted to simulate the search of a typical user looking for images of infant sleep environments (eg, an expectant parent searching for nursery ideas) by using hashtags. Because images in magazines, advertisements, and the internet are often inconsistent with these guidelines [42-44], we hypothesized that the majority of images on Instagram for infant sleep–related hashtags, extracted through a web scraper (which uses automated processes to gather specific data from a website [57]), would also be inconsistent with infant safe sleep guidelines, as published by the AAP [27], and that the number of “likes” for each image would not correlate with adherence of the image to these guidelines.

## Methods

We conducted a search for images on Instagram using hashtags that were relevant to infant sleeping practices and environments (as might be done by someone looking for ideas for nursery products). These hashtags were determined by conducting an initial cursory search on Instagram; the hashtags that yielded the greatest frequency of relevant searches were used. Images had to contain a sleeping infant or a sleep environment that appeared to be intended for an infant. Sleep locations not solely intended for infant use (eg, beds, sofas) were included only if a sleeping infant was present. The data were collected via an open-source web scraper (provided by user jaroslavejhlek) on the data scraping website Apify [58].

The first 200 images from each hashtag were utilized for this analysis, as we believed it to be unlikely that users would look beyond the first 200 images in a typical search. All images were either photographs or video thumbnails (still images that preview videos) and were preliminarily sorted into groups that either depicted a sleeping infant or a sleep environment without an infant. Afterward, they were analyzed more thoroughly for adherence to AAP safe sleep guidelines. Table 1 presents the scoring criteria.

**Table 1:** Criteria for images.

Category	Consistent with AAP^a^ guidelines	Inconsistent with AAP guidelines
Sleep position	Supine, held by an awake adult	Side, prone, sitting or upright, held by a sleeping adult
Sleep location	Crib, portable crib, play yard, bassinet, Moses basket, bedside co-sleeper, ground; sleep surface horizontal; no cushioning of sides	Bed (any size); sitting device (car seat, swing); couch, sofa, armchair; in-bed co-sleeper, positioner, or infant “dock” (eg, DockATot); sleep surface not horizontal; sides of sleep product (if applicable) are cushioned
Bedding	No bedding in the sleep area	Presence of unswaddled blanket, pillow, bumper, plush toys, or other soft bedding
Bed-sharing	Infant is not on the same sleep surface as another person or animal	Infant is on the same sleep surface as another person or animal
Head covering	No head covering	Head covering of infant
Strangulation risk	No strangulation risks	Strangulation risks (eg, long ties, drapes)

^a^AAP: American Academy of Pediatrics.

Each image was analyzed by 2 authors, and any discrepancies were reconciled by a third. Images were categorized as consistent with AAP safe sleep guidelines if the sleep surface appeared to be firm and flat (horizontal), without any soft bedding or strangulation risks; if a sleeping infant was visible, the infant had to be supine or held by an awake adult and could not be wearing a head covering.

The number of “likes” associated with each picture at the time of scraping was also collected. Statistical analysis included descriptive statistics. Unpaired *t*-tests, assuming unequal variances, were conducted to determine whether the number of likes was associated with whether the image depicted a safe sleep environment. Because this study involved the collection and study of publicly available data, it was considered exempt by the Institutional Review Board of the University of Virginia.

## Results

### Overview

Data from 27 hashtags were collected in June 2020. Of the 5400 Instagram images scraped (first 200 images from 27 hashtags), a total of 1563 met inclusion criteria. Of those, nearly three-quarters (1134, 72.55%) had a sleeping infant, and 429 (27.45%) portrayed a sleep environment without a sleeping infant (Table 2). Of the 1563 images, 117 (7.49%) were consistent with AAP safe sleep guidelines. For another 93 (5.95%) images, the sleep location (eg, crib, bed) of the sleeping infant could not be determined, but these images otherwise were consistent with AAP safe sleep guidelines.

Table 3 provides details about the images. The percentages in Table 3 are row percentages, which indicate the number of images in the particular cell, divided by the total number of images in the same row.

**Table 2:** Instagram hashtags included in analysis.

Hashtag	Total images (N=1563), n	Images consistent with AAP^a^ guidelines, n (%)
#baby	11	0 (0)
#babynursery	33	2 (6)
#babynurserydecor	22	4 (18)
#babyshowergiftideas	7	0 (0)
#babysleep	49	2 (4)
#babysleeping	59	4 (7)
#bassinet	83	29 (35)
#crib	61	21 (34)
#cutebabiesofinstagram	23	0 (0)
#infantphotography	22	0 (0)
#infantsleep	43	1 (2)
#naptime	13	0 (0)
#newborn	67	1 (1)
#newbornbaby	74	1 (1)
#nursery	22	2 (9)
#nurserydesign	36	3 (8)
#nurseryinspiration	63	8 (13)
#nurseryinspo	33	0 (0)
#projectnursery	123	28 (23)
#safebaby	25	0 (0)
#sleepbaby	52	2 (4)
#sleepingbabyboy	123	0 (0)
#sleepingbabygirl	129	0 (0)
#sleepingbabyphotography	115	0 (0)
#sleepinginfant	125	3 (2)
#Sleepybaby	76	0 (0)
#twoweeksold	74	4 (5)

^a^AAP: American Academy of Pediatrics.

**Table 3:** Characteristics of images.

Category				Total (n=1563), n	Bedding present, n (%)^a^	Bed-sharing, n (%)	Posed^b^, n (%)	No baby present, n (%)	Location unknown, n (%)
Images consistent with AAP^c^ guidelines				117	0 (0)	0 (0)	0 (0)	95 (81.20)	0 (0)
Images inconsistent with AAP guidelines				1446	1173 (81.12)	66 (4.56)	167 (11.55)	332 (22.96)	572 (39.56)
Images with sleeping infant present				1134	845 (74.51)	66 (5.82)	174 (15.34)	0 (0)	662 (58.38)
Images with no sleeping infant present				429	328 (76.46)	0 (0)	0 (0)	429 (100)	1 (0.23)
**Images with infant in sleep position inconsistent with AAP guidelines**				479	387 (80.79)	28 (5.85)	122 (25.47)	0 (0)	254 (53.03)
		Supine		488	406 (83.20)	31 (6.35)	45 (9.22)	0 (0)	299 (61.27)
		Side		222	192 (86.49)	16 (7.21)	36 (16.22)	0 (0)	127 (57.21)
		Prone		156	131 (83.97)	10 (6.41)	52 (33.33)	0 (0)	27 (17.31)
		Sitting/upright		101	64 (63.37)	2 (1.98)	34 (33.66)	0 (0)	27 (26.73)
		Held by an awake adult		169	54 (31.95)	7 (4.14)	7 (4.14)	0 (0)	110 (65.09)
**Images of an infant sleep location inconsistent with AAP guidelines**				162	103 (63.58)	12 (7.41)	18 (11.11)	1 (0.62)	0 (0)
	Crib			422	326 (77.25)	2 (0.47)	5 (1.18)	333 (78.91)	0 (0)
	Bassinet/Moses basket			150	113 (75.33)	1 (0.67)	5 (3.33)	82 (54.67)	0 (0)
	Bed (any size)			128	116 (90.63)	31 (24.22)	18 (14.06)	0 (0)	0 (0)
	Sitting device			90	47 (52.22)	1 (1.11)	14 (15.56)	0 (0)	0 (0)
	Ground			31	20 (64.52)	2 (6.45)	4 (12.90)	1 (3.23)	0 (0)
		Couch/sofa/cushioned armchair		55	40 (72.73)	11 (20.00)	4 (7.27)	0 (0)	0 (0)
		In-bed co-sleeper, positioner, or dock		17	16 (94.12)	0 (0)	0 (0)	1 (5.88)	0 (0)
		Location unidentifiable		670	491 (73.28)	18 (2.69)	124 (18.51)	1 (0.15)	670 (100)
**Images of sleeping infant on the same surface as another sleeping person or animal**				65	60 (92.31)	65 (100)	4 (6.15)	0 (0)	18 (27.69)
		Sharing with adult		37	33 (89.19)	37 (100)	1 (2.70)	0 (0)	6 (16.22)
		Sharing with child		21	20 (95.24)	21 (100)	3 (14.29)	0 (0)	8 (38.10)
		Sharing with animal		7	7 (100.00)	7 (100)	0 (0)	0 (0)	3 (42.86)
**Images with bedding present**				1173	1173 (100)	61 (5.20)	139 (11.85)	319 (27.20)	491 (41.86)
		Unswaddled blankets		832	832 (100)	45 (5.41)	95 (11.42)	177 (21.27)	376 (45.19)
		Bumpers		146	146 (100)	2 (1.37)	7 (4.79)	82 (56.16)	15 (10.27)
		Pillows		536	536 (100)	34 (6.34)	62 (11.57)	185 (34.51)	187 (34.89)
		Other bedding		331	331 (100)	9 (2.72)	54 (16.31)	114 (34.44)	126 (38.07)
Images with infant head covered				191	156 (81.68)	6 (3.14)	55 (28.80)	0 (0)	12 (6.28)
Images with potential strangulation risk				196	162 (82.65)	8 (4.08)	80 (40.82)	0 (0)	140 (71.43)
Images with swaddled infant				116	94 (81.03)	9 (7.76)	41 (35.34)	0 (0)	75 (64.66)
Images with posed infant^b^				174	139 (79.89)	4 (2.30)	174 (100)	0 (0)	123 (70.69)
Images with pacifier				98	69 (70.41)	7 (7.14)	3 (3.06)	0 (0)	52 (53.06)

^a^All percentages are row percentages, with the total images in that category as the denominator.

^b^Images with posed infant refer to images of infants that were obviously posed and did not represent true sleep environments (eg, flowerpots).

^c^AAP: American Academy of Pediatrics.

### Position

Of the 1134 images that portrayed a sleeping infant, 488 (43.03%) showed the infant sleeping supine, and 169 (14.90%) showed a sleeping infant held by an awake adult. There were 479 (42.24%) images that were inconsistent with AAP recommendation to place infants supine on a firm and flat surface, including 222 (19.58%) images with an infant sleeping on the side, 156 (13.76%) with an infant sleeping prone, and 101 (8.91%) with a sleeping infant that was in the sitting position.

### Bedding

In the 1563 images of infant sleep environments, the presence of bedding was the most common reason that the image was inconsistent with safe sleep guidelines; of all images, 1173 (75.05%) contained some form of soft or loose bedding. The most commonly observed bedding type was an unswaddled blanket, which was present in 836 images (71.27% of 1173 images with bedding, 53.49% of all 1563 images). The next most common was a pillow, found in 536 images (45.69% of 1173 images with bedding, 34.29% of all 1563 images). Bumpers were found in 146 images (12.45% of 1173 images with bedding, 9.34% of all 1563 images), and a stuffed animal or other soft bedding was found in 331 images (28.22% of 1173 images with bedding, 21.18% of all 1563 images).

### Location

A crib, bassinet, play yard, or bedside co-sleeper was the most commonly observed sleep location (422/1563, 27.00%). Other sleep locations included a Moses basket (150/1563, 9.60%); adult or child bed (128/1563, 8.19%); sitting device such as a car seat or stroller (90/1563, 5.76%); a couch or sofa (55/1563, 3.52%); the ground or floor (31/1563, 1.98%); and an in-bed co-sleeper, positioner, or infant “dock” (eg, DockATot; 17/1563, 1.09%). The largest proportion (670/1563, 42.87%) of images portrayed an infant in a location that could not be definitively identified. Of the images with an unidentified location, 577/670 (86.1%) demonstrated other aspects of the sleep environment that were inconsistent with AAP safe sleep guidelines.

### Bed-sharing

Bed-sharing was seen in 65 (5.73%) of all 1134 images with an infant present. An adult bed-sharer was the most common (n=37; 22.4% [37/165] of bed-sharing images, 3.26% [37/1134] of images with an infant present) followed by a child (n=21; 12.7% [21/165] of bed-sharing images, 1.85% [21/1134] of images with an infant present) and an animal (n=7; 4.24% [7/165] of bed-sharing images, 0.62% [7/1134] of images with an infant present).

### Posed Images

We separately analyzed images in which the infant was obviously posed and did not represent a true infant sleep environment (eg, flowerpot). This category does not include other images for which the infant may have been posed but were potential infant sleep environments (eg, infant posed on a sofa). There were 174 such images (15.34% of 1134 images with an infant present). Of these, 167 (96.0%) images had elements that were inconsistent with AAP guidelines. The other 7 images (4.0%) were consistent with AAP guidelines with the possible exception of the sleep location, which could not be determined. In the 174 posed images, the infant was prone in 52 (29.9%), supine in 45 (25.9%), on the side in 36 (20.7%), sitting upright in 34 (19.5%), and held by an adult in 7 (4.0%). Infants in posed images, when compared with those in unposed images, were 19.7 percentage points more likely to be portrayed in a nonsupine or upright position (*P*<.001), and were overall more likely to be portrayed in a sleep environment that was inconsistent with AAP guidelines (*P*<.01).

### Likes

Images adhering to AAP safe sleep guidelines had a mean 127.8 likes (SD 370.5); if the images with undetermined location were excluded, the mean like count was 181.7 (SD 461.0). Images with elements inconsistent with AAP guidelines had a mean of 128.4 likes (SD 509.9). There was no significant difference in the mean like count between nonadherent images and total adherent images (*P*=.99). When images with undetermined location were excluded, images adhering to AAP guidelines had a higher mean like count than nonadherent images (*P*=.001).

## Discussion

### Principal Findings

Of the 1563 Instagram images analyzed, only 117 (7.49%) were clearly consistent with AAP safe sleep guidelines. Another 93 (5.95%) were possibly consistent, but were taken in such a way that the sleep environment could not be fully visualized. This means that, even when these images with incomplete information are included, an overwhelming majority (1353/1563, 87%) of Instagram images portrayed unsafe infant sleep environments, as defined by the AAP.

Nearly half of businesses are active on Instagram [56]. As with any marketing strategy, businesses use Instagram to increase sales of their product(s). Businesses are guided to post aesthetically pleasing photos of their products, liberally use hashtags, and facilitate purchases from the website [59]. Company statistics indicate that these strategies are successful in promoting sales of products, as nearly half of surveyed Instagram users stated that they have purchased a product after seeing it on Instagram [56]. While Instagram’s Community Guidelines prohibit content with “the potential to contribute to real-world harm” [60] and the Commerce Policies prohibit sale of “medical and healthcare products and services, including medical devices” [61], there are no rules that specifically address posting of photos that demonstrate unsafe sleep practices.

Although many prospective and new parents purchase products from traditional stores that sell products in person (and in some stores, employees may provide guidance regarding safe sleep guidance), there has been a steady increase over the past decade in the proportion of products sold online [62]. Thus, images posted online, particularly on websites that are viewed by a large proportion of the population, can be extremely influential [63]. Many companies, especially those that advertise on Instagram, utilize a “brand ambassador” program in which parents themselves are sponsored to post pictures promoting a certain product. A direct potential consequence of peers consistently modeling and posting images of specific, unsafe sleep environments is the misconception among new parents that these practices are safe, when physician advice is to the contrary. With regard to infant sleep practices, mothers are more likely to change from safe to unsafe sleep practice if their network members substantially espouse unsafe practices [64], and this may be true for virtual network members as well. The ability for social media to influence the behavior of a large proportion of the population is a well-known phenomenon [47]; indeed, there are now “influencers,” who are especially prominent on Instagram. These individuals are paid for their posts because their use of products results in increased sales [65]. Not only do they influence certain practices, but they may create completely new ones as well [66,67].

The Theory of Planned Behavior states that one’s behavior is shaped by social norms, and that these norms directly impact one’s attitudes about the specific behavior [68]. One’s practices and rationalizations for these practices are learned from and reinforced by others [69,70], so that one’s behavior becomes increasingly similar to that of network members [2,3,15-22]. Infant sleep practices, especially sleep environments, are not immune from these forces. Images of cribs and bassinets littered with toys, blankets and pillows, infants sleeping nonsupine, or infants wearing warm head coverings or hats with long strings (that pose a strangulation hazard) are displayed, often unopposed, on social media sites. The images, which are usually well produced and chosen because they appear “authentic,” come to represent “desirable” environments one wants to emulate [71]. With no regulations relevant to safe infant sleep practices inherent to Instagram, the proxy for acceptability may become how popular or common a sleep environment is. Even though not all of the nonadherent images were posted by individuals, and many (174/1134, 15.3%) were very obviously posed for a photographer, our findings demonstrate that the culture of infant sleep on Instagram is one that does not promote infant safety as a priority.

Nearly half (479/1134; 42.24%) of sleeping infants were portrayed in the nonsupine position; while some may think it encouraging that the majority were in recommended positions (supine or held by an awake adult), the sizeable proportion of infants sleeping nonsupine suggests that supine positioning is not the social norm for many. More concerning is the majority (1173/1563; 75.05%) of images demonstrating the presence of soft bedding. This proportion is similar to national data on bedding use reported by Shapiro-Mendoza and colleagues [37]. Important reasons for such widespread use of soft bedding by parents or guardians include concerns about comfort and warmth [72]. On a social media platform such as Instagram, the use of bedding could also be for purely aesthetic purposes [72], or to signify the status or creativity of the person posting the image.

Cribs were found in 77.62% (333/429) of the images with no baby present, but in only 27.00% (422/1563) of images overall. Many of these images were posted by marketers or decorators (eg, #nurseryinspirations) who aim to establish images of expected or normative practice for parents decorating a new nursery. While cribs are consistent with AAP safe sleep recommendations, many of the other products shown in these marketing images are not. Three-quarters (1173/1563; 75.05%) of these images had soft bedding, including loose blankets, pillows, and bumpers, present.

There is a common assumption that if an object is being sold, then it is safe to use [44]. On social media platforms, the safety assumption can be taken one step further because there is a scoring mechanism to see how popular a practice is: likes. Pictures with more likes may be viewed as more acceptable and thus safe. With regard to the number of likes for images that were consistent or inconsistent with AAP safe sleep guidelines, the mean was similar for these 2 categories. However, it should be noted that there were approximately 12 times fewer images that were consistent with safe sleep guidelines, potentially creating a bias.

### Limitations

This study, as is any study involving social media as its data source, is limited by the fact that the sample is inherently biased. Individuals who use Instagram and post pictures of sleeping infants or infant sleep environments may not actually use these practices regularly. For example, a large proportion of images included bedding. Blankets, stuffed animals, and pillows can all be used to make the image more aesthetically pleasing, but may not be in the infant’s actual sleep environment. However, the purpose of this study was not to analyze actual practices, but to look at the culture of what is considered desirable to display and be propagated on the platform. We also did not analyze image captions, which may alter the viewer’s perception of the image. However, Tiggemann et al [73] found that the effect of a “positive” caption did not significantly change someone’s perception of an image that would otherwise make them feel dissatisfied with their body. Further study into the types of captions associated with certain hashtags, as well as the content of captions in safe versus unsafe pictures is necessary to more fully understand the landscape of safe infant sleep on Instagram.

### Conclusions

In conclusion, the vast majority of images pertaining to infant sleep are inconsistent with AAP safe sleep guidelines. It is imperative that health care providers at least know and understand the landscape of normative practices on social media so they can best tailor either specific patient advice or public health approaches [74]. Additionally, campaigns to promote safe sleep may require health care professionals and officials to work with influencers and social media companies to promote up-to-date, evidence-based information about current recommendations that is trustworthy and engaging.

## Abbreviations

AAP: American Academy of Pediatrics
SUID: sudden unexpected infant death
